# Protein corona and exosomes: new challenges and prospects

**DOI:** 10.1186/s12964-023-01089-1

**Published:** 2023-03-27

**Authors:** Morteza Heidarzadeh, Amir Zarebkohan, Reza Rahbarghazi, Emel Sokullu

**Affiliations:** 1 Koç University Research Centre for Translational Medicine (KUTTAM), Koç University School of Medicine, Istanbul, Turkey; 2 Department of Medical Nanotechnology, Faculty of Advanced Medical Sciences, Tabriz University of Medical Sciences, Tabriz, Iran; 3 Department of Applied Cell Sciences, Faculty of Advanced Medical Sciences, Tabriz University of Medical Sciences, Tabriz, Iran; 4 Stem Cell Research Center, Tabriz University of Medical Sciences, Tabriz, Iran; 5 Biophysics Department, Koç University School of Medicine, Rumeli Feneri, 34450 Sariyer, Istanbul, Turkey

**Keywords:** Exosomes, Protein corona, Physicochemical properties, Biodistribution

## Abstract

Recent advances in extracellular vesicle (EVs) detection and isolation methods have led to the development of novel therapeutic modalities. Among different types of EVs, exosomes (Exos) can transfer different signaling biomolecules and exhibit several superior features compared to whole-cell-based therapies. Therapeutic factors are normally loaded into the Exo lumen or attached to their surface for improving the on-target delivery rate and regenerative outcomes. Despite these advantages, there are several limitations in the application of Exos in in vivo conditions. It was suggested that a set of proteins and other biological compounds are adsorbed around Exos in aqueous phases and constitute an external layer named protein corona (PC). Studies have shown that PC can affect the physicochemical properties of synthetic and natural nanoparticles (NPs) after introduction in biofluids. Likewise, PC is generated around EVs, especially Exos in in vivo conditions. This review article is a preliminary attempt to address the interfering effects of PC on Exo bioactivity and therapeutic effects.

**Video Abstract**

**Graphical Abstract:**

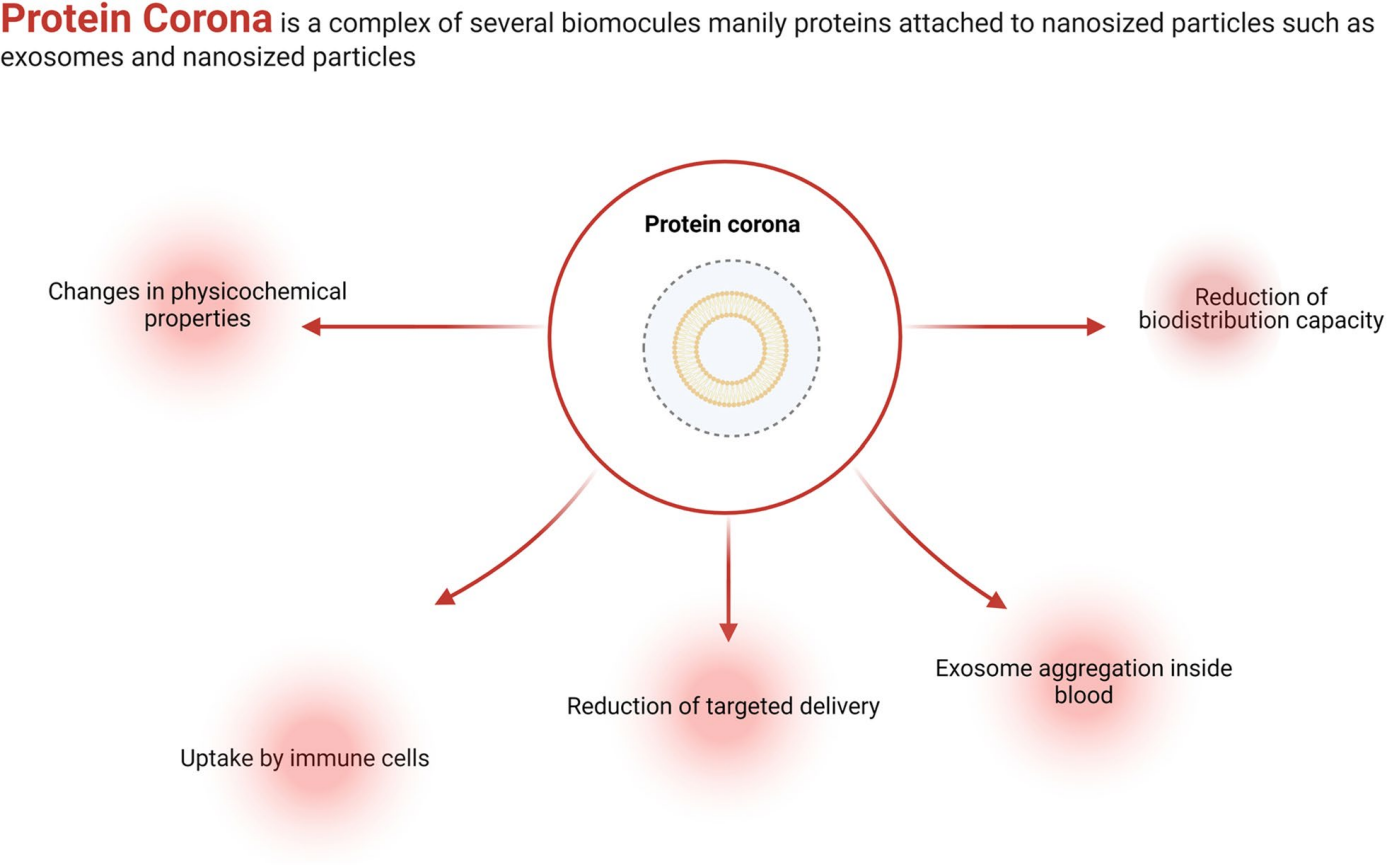

**Supplementary Information:**

The online version contains supplementary material available at 10.1186/s12964-023-01089-1.

## Background and Exo biogenesis

Exo therapy is touted as a fascinating field with significant therapeutic potential in patients compared to numerous conventional modalities and whole-cell therapy [1]. These beneficial outcomes can be increased and fine-tuned via the loading of specific cargo and surface modification in favor of an increased target delivery rate [2]. Indeed, Exos are nano-sized particles (50–150 nm) belonging to extracellular vesicles (EVs) and involved in paracrine cell-to-cell connection [3]. It is thought that the endosomal system, composed of early, and late endosomes, and mature multivesicular bodies (MVBs), is the main elaborate intracellular pathway for the generation and abscission of Exos from parent cells [4]. Ultrastructural analyses have shown that Exos are originated from intraluminal vesicles (ILVs) following the invagination of lipid membrane into the lumen of late endosomes and MVBs via the activity of the endosomal sorting complex required for transport (ESCRT) complex [5]. Four ESCRT subsets, including ESCRT-0, -1, -II, and -III can orchestrate the invagination process and cargo sorting [6]. It is thought that cargo sorting is done via the direct recognition and interaction of ubiquitinated components with binding sites located at ESCRT-0. The process continues with the attachment of ESCRT-I and -II to the ESCRT-0. With the addition of ESCRT-III, the phenomenon of invagination is initiated and ILVs are pushed into the lumen of MVBs [7]. After the completion of ILV formation, Vps4 can separate ESCRT-III from the ESCRT complex and stop the invagination process. Other factors belonging to the ESCRT complex such as Alix, TSG101, and CHMP4 can be involved in ILV budding and subsequent abscission [4]. Like the ESCRT-dependent pathway, ESCRT-independent mechanisms such as raft-based microdomains with sphingomyelinase activity and ceramides are alternates to induce cargo segregation and ILV budding. Tetraspanins (CD9, CD9, and CD63) are other molecular machinery that participates in protein sorting and ILV formation [7]. In the latter steps, the direct fusion of MVBs with plasma membrane releases ILVs into the ECM where they are so-called Exos [8]. The subsets of the SNARE family are involved in the docking and fusion of MVBs with the plasma membrane [9]. SNARE proteins are classified as t- and v-SNAREs with the potential to accelerate the process of fusion in direct collaboration with Ras proteins. Along with these factors, Rab family proteins can stimulate MVB tethering via the promotion of interaction with cytoskeletal elements [10]. Upon the activity of cargo sorting system, varied biomolecules such as peptides and genetics are sequestrated into the ILVs lumen [11]. Based on numerous molecular investigations, Exo entry into the target cells relies on the solo performance or simultaneous activity of several mechanisms including phagocytosis, macropinocytosis, and clathrin-based endocytosis [6]. For instance, cholesterol lipid rafts can pave a way for Exo uptake in acceptor cells under specified circumstances [12]. How and which of these mechanisms dominate in Exo recognition and uptake during physiological and pathological conditions are the subject of debate.

Here, we tried to collect recent data related to the possible impacts of PC on Exo activity in in vitro and in vivo milieu. These data can help us to understand whether the formation of PC around Exos can affect normal activity inside the body.

## PC formation around nano-sized particles

Despite recent progress in surface modification techniques and transplantation of NPs, it has been indicated that any ex vivo manipulations can affect the  delivery rate and therapeutic outcomes [13]. The close interaction with the set of proteins and factors leads to the formation of a proteinaceous layer on the NP surface in biofluids. This layer, known as PC, is generated via engaging several mechanisms [13]. Because of similarities between synthetic NPs and Exos in terms of size, dimensionality, and active surface, it is logical to hypothesize the formation of PC could in part, but not completely, affect the dynamic activity of Exos [14]. According to recent data, the formation of PC around nanoscale biomaterials can change the fate of signaling cargoes inside in vivo conditions [15]. Irrespective of the substantial differences between synthetic NPs with other nano-sized particles such as Exos and viruses, common aspects like similar size and dimensionality can affect their biological effects [16, 17].

The formation of PC around the NPs depends on two main parameters. First, physicochemical values like size and diameter, surface curvature and entropy, lattice parameters, and net charge affect the possibility of PC formation. Second, the existence of specific surface proteins, receptors, and biological molecules can increase the possibility of PC formation [13, 18]. It was suggested that a set of substrates in a stochastic environment can also result in PC formation. To be specific, there is a close relationship between metabolite content and collision frequency [13, 18]. The term collision frequency is associated with an average atomic interaction (collision) of two reactants or molecules per unit of time at a specific aqueous system [19]. It is estimated that an average collision frequency is about 10^6^/sec in the blood [13, 18]. Interestingly, the formation of PC can immediately alter the size, hydrophobicity/hydrophilicity ratio, zeta potential, and the surface fingerprint of NPs within the first 30 s following distribution in the circulation system [13, 18]. The type and amount of adsorbed biomolecules can significantly alter these values [20]. It should be noted that energetics of adsorption and desorption pre-determine the efficiency of collision frequency and thus PC formation around NPs. In better words, the propensity of specific protein types toward specific surfaces depends on the equilibrium dissociation constant and Gibbs free energy value [13]. Surface hydrogen bonding, hydrophobic interactions, and van der Waals forces are also involved in the affinity of proteins and bio-compounds around NPs [21]. Following the entry of NPs into biofluids, these forces adsorb free proteins to minimize free enthalpy and thermodynamic forces [21]. Upon the binding of proteins to the NPs surface, their hydration layer is spatially displaced due to increased entropy because of energetic protein binding and reduced enthalpy [22]. According to Vroman’s effect, small-sized and more concentrated proteins attach to the NP surface in early steps after being introduced into the blood. These proteins can be replaced by less-content but high-affinity proteins by time [22–24].

## Protein corona components

Numerous investigations have revealed two distinct layers, including inner hard and outer loose corona layers after the adsorption of low and high-affinity proteins around NPs [25]. It is suggested that the entity of the PC is changed over time because of alterations in the composition of the hard PC layer [26]. Molecular investigations have revealed that PC formation is done in different phases around the silica microparticles and irreversible absorption of proteins leads to the formation of the inner hard PC layer. In the latter steps, the reversible interaction of low-affinity proteins results in the formation of the outer soft PC layer [27]. The nature and composition of the soft PC layer are constantly changed under flow conditions [28, 29]. Interestingly, the protein–protein interaction is mainly involved in the soft corona formation because the surface of the NPs pre-occupied with the hard PC layer. *In-situ* investigations have indicated the axis role of soft corona compositions on the stealth properties of the liposomes [25]. This feature correlates with the absorption of different molecules in biofluids and the physicochemical properties of NPs. Commensurate with these descriptions, the composition, and levels of proteins in the hard corona layer can reflect the identity of biomolecules under physiological and pathological conditions [30].

## The component of PC is based on NP properties

In terms of PC formation, the physicochemical properties of NPs should not be neglected. The decoration of NPs with PC layers especially the inner hard PC can lead to the activation of the reticuloendothelial system cells and the elimination of NPs faster than the expected time [31, 32]. This effect is simultaneously intensified by the accumulation of misfolded proteins and NP aggregation. In recent years, many efforts have been collected to profile the  molecular composition of the PC. Using immunoblotting and gel electrophoresis techniques, Cullis and co-workers identified several PC subsets around liposomes that are associated with the liposomes' half-life [33]. In an experiment, more than 300 different factors have been recognized around gold NPs (AuNPs) [34]. Using LC–MS/MS and ELISA analyses, about 288 serum proteins were detected around AuNPs and interestingly 93% of PC components are generated by 80 proteins [35]. Based on ELISA data, 87% of PC compositions are anti-thrombin III, complement C3, factor V, fibronectin, IgG, and complement factor H [35]. Thus, these findings show that most serum proteins do not participate in PC formation because of a weak binding capacity. If so, the majority of these proteins do not lose their bioactivity even after adsorption onto the NP surface. On the other hand, the formation of the inner hard PC layer restricts the further recruitment of other plasma proteins [35]. According to recent findings, there is a close relationship between AuNP type and aggregation of distinct serum proteins. For example, proteins such as plasminogen, β-globulin, or serine protease inhibitor A3N, apolipoprotein A-I, and murinoglobulin-2, have unique tendencies for gold nanorods and nanostars, respectively [30]. The size, surface chemistry, shape, and entropy of NPs are the most important parameters in plasma protein adsorption thus biodistribution capacity [30]. Within biological samples, the incubation of NPs with similar surface chemistry and composition but different sizes can contribute to the formation of PC ranging from 30 to 200 nm in diameter. In larger particles with an average diameter of more than 400 nm, there is no close association between the type of PC and NP size [36]. Noteworthy, the alteration of the surface composition can profoundly change the PC content around NPs [37]. It means that the finding of a common rule in the PC phenomenon is not available soon due to the effect of many unknown parameters. In the other words, decorating the surface of NPs with any type of ligands such as small molecules, peptides, aptamers, proteins, antibodies, etc. other parameters like density, molecular weight, chain length, etc. should be addressed in detail in terms of PC formation. The role of the administration route (intravenously, orally, and inhalation) is also critical in the composition and profile of PC. Any changes in PC content are directly associated with environmental characteristics such as the velocity of blood flow, laminar/non-laminar blood flow, sex (gender), and temperature [38]. It has been found that the stability of PC, especially the soft corona layer, can be changed based on environmental properties like blood flow velocity from capillaries to arteries. The occurrence of pathological conditions in the vascular wall such as aneurysms can also alter the entity of the soft corona layer. In this regard, faster fluids with high concentrations of plasma proteins lead to less NP aggregation [39]. Possibly, the rational reason behind this phenomenon is related to the chaos of the blood component's random thermal motion by increasing the speed of blood. Noteworthy, NPs are directly faced with thousand  protein types but hundreds of different proteins are intended to involve in reciprocal protein-surface interactions due to the restricted available area after the first layer formed. In this scenario, the binding of varied proteins to floated NPs may lead to the formation of different NP subpopulations that consequently affect biological activity in in vivo conditions [27].

Interestingly, the certain proteomic profile of each pathological condition is another indisputable factor affecting the PC content around specific NPs [40]. For instance, Mahmoudi et al. indicated that PC entity around the pristine polystyrene NPs depends on lifestyle, pregnancy, thalassemia, hypertension, cancers, etc. It should not be neglected that PC formation is a protein concentration-dependent process, as this phenomenon started from 10% protein in the biological fluids [41]. Surprisingly, this profile differs individually in the same condition (healthy or diseased persons). Graphene is a carbon-based nanomaterial composed of several carbon sheets with higher carrier mobility, elasticity, and surface-to-volume ratio. Graphene is commonly used in tissue engineering and drug delivery due to its therapeutic applications [40]. After incubating graphene oxide sheets with serum or plasma of patients with major thalassemia, and plasma from cancer patients, the production of NO and ROS increased compared to the control group [40].

## Formation of PC on the surface of Exos

**Fig. 1:**
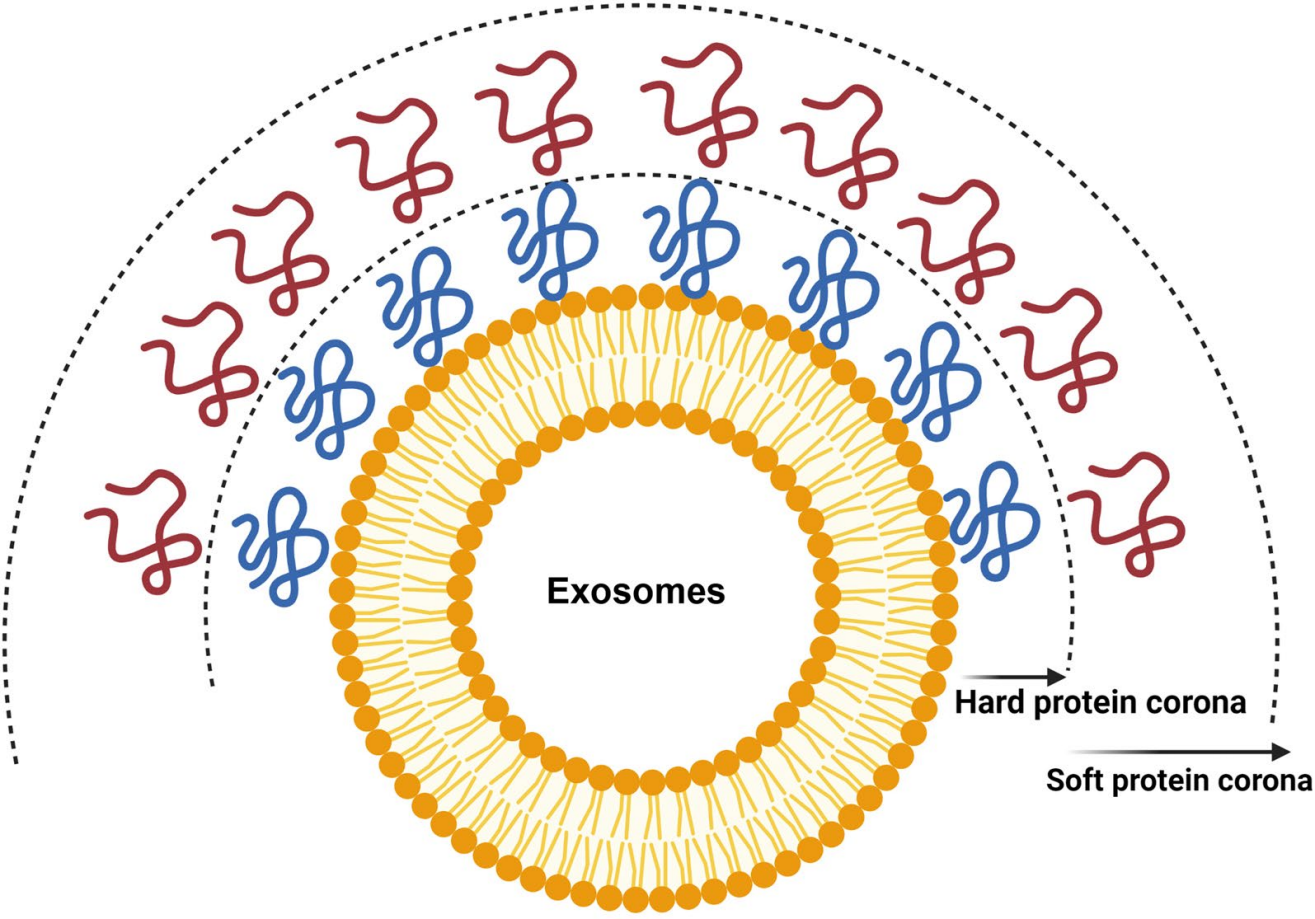
Formation of PC around Exos after exposure to biofluids. PC consists of external soft and internal hard layers

Emerging pieces of evidence have shown the formation of PC around Exos inside aqueous phases via electrostatic interactions and protein aggregation [14] (Fig. 1). The mechanisms related to the close interaction of viruses and host systems need to be re-examined concerning PC formation. Of course, the lack of suitable knowledge about factors participating in PC formation within biofluids has led to theoretical and experimental immaturity. Notably, there are some scientific documents associated with PC formation around nano-sized particles such as viruses [42]. It is thought that the existence of a unique 3D conformation structure and certain types of amino acids around viral capsids or envelopes can lead to the weakest mode of mutual interaction between viruses and soluble proteins. These features can lead to the formation of a loosely soft PC layer on the surface of circulating viruses [43, 44]. Upon the attachment of viral ligands to cell surface receptors, the soft PC layer is easily separated in nanoseconds and the virus ultimately enters the host cells [45–47]. The presence of viral inclusion bodies in certain neurological diseases like Alzheimer's disease and multiple sclerosis was unclear for a long time [48, 49]. Interestingly, it is suggested that protein fibrillation can be initiated following nano-bio interaction (PC formation), leading to an impaired 3D protein structure. In line with this claim, Ezzat et al. showed the formation of PC around some virus types such as HSV1 [16, 50]. Likewise, the close interaction between SARS-CoV-2 virus plasma proteins can alter ApoE confirmation and exposure of binding epitopes to the immune cells [51]. Due to limited information related to the effect of PC on the dynamic activity of viral particles, one can hypothesize that differences in human proteomics can, in part but not completely, affect the pathogenicity of viruses via blunting properties of PC. As such, the existence of surface charge and other parameters such as size diversity and several surface biomolecules, the continuous contact of Exos with plasma and interstitial fluids can result in the absorption of protein arrays [52]. It is thought that ligand-receptor affinity is another mechanism involved in the accumulation of external protein on the Exos surface [53].

## Effects of PC on Exo activity

Surface interaction of circulating Exos with different soluble factors can affect exosomal tropism and delivery capacity into the targeted lineage cells (Table 1). Due to the similarity in the size of Exos with NPs, and viruses, a protein-rich layer decorates Exos upon exposure to blood plasma [14]. It is thought that incubation with plasma samples is the best strategy for the study of PC formation and simulation of NP behavior in in vivo circumstances because of the varied protein contents present in blood [54, 55]. In situ contact of Exos with the tumor microenvironment (TME) can lead to the attachment of different types of tumor-related cytokines, metabolites, and distinct proteins which can be found relatively specific in pathological sites such as TME [53]. An increased MDR activity is associated with a stimulated membrane-derived vesicle recycling rate, leading to lower intracellular drug concentration and anchorage lipid rafts. As a correlate, these cells are resistant to cell death receptors activity and apoptotic changes [56–58]. Exos derived from these cells exhibit different binding capacities with specific PC entities rather than that of normal counterparts. Under such circumstances, the rigidity and fluidity of the cell membrane are sensitive to the fluctuation of ambient temperature and pH values. To be specific, lower pH indices can increase the fusogenic properties of cell membrane lipids and cause lipid inter-digitation as an important process during exosomal fusion [59, 60].

**Table 1:** Some PC components adsorbed around Exo

Hard and Soft PC	Type of interactions	Effects on EVs	References
Albumin	Connected to RNA/DNA residues on the Exo	Aggregation, phagocytosis of Exo	[75, 76]
Complement factors 3	Interaction with CD63 on the Exo surface	Increases robust phagocytic activities Triggers secondary inflammatory reactions	[14]
Apolipoprotein A1	Interaction with CD63 on the Exo surface	Phagocytosis	[14]
Apolipoprotein B	Interaction with CD63 on the Exo surface	Increases robust phagocytic activities Triggers secondary inflammatory reactions	[14]
Apolipoprotein C3	Interaction with CD63 on the Exo surface	Short-time biodistribution and reduction of transit time Phagocytosis of Exo	[14]
E, α component of fibrinogen	Protein–protein interactions	Short-time biodistribution and reduction of transit time Protein and Exo aggregation	[14]
immunoglobulin heavy chains of (γ2 and γ4)	Protein–protein interactions	Short-time biodistribution and reduction of transit time	[14]
Complement proteins C3b and C3ib	Protein–protein interactions	Prolonged chronic inflammatory conditions	[89]
ApoE and ApoB100	Protein–protein interactions	Increase the transfer rate into the peripheral tissues	[97, 98]
S100-A8, LDL-receptor, CD14, HLA class I	Phosphatidylserine and tissue factor on the Exo surface	Dynamic activity	[102, 103]
VII, IX, X/prothrombin,	Tendency to tissue factors	Contribute to thromboembolic complications	[133]
Mismatched MHC-I and II	On the surface of Exo	T cell-related immune responses	[112]

**Fig. 2:**
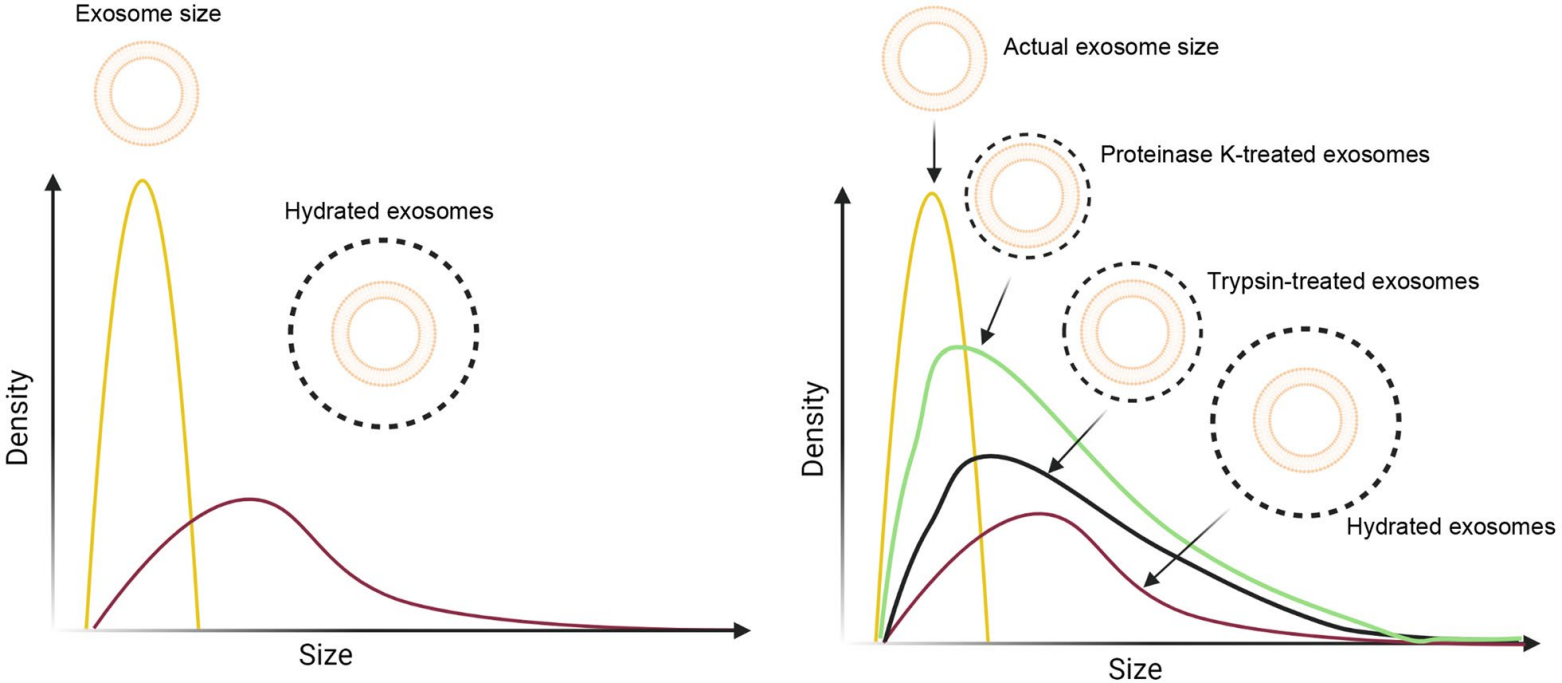
Exo hydrodynamic size is increased after PC information. Reduction of exosomal size after exposure to trypsin and especially proteinase K indicates a large number of soluble proteins attached to the Exo surface

In short, because of the intricate structure and existence of several ligands on the exosomal surface, the biodistribution and the fate of Exos are differently affected by PC formation in contrast to simple NPs structures. It is hypothesized that any particles are composed of "N" components participating in the net resultant force field. To be specific, NPs can sense surrounding media using reciprocal short/long-range physical force fields [61, 62]. Therefore, surface modification of Exos with small molecules and components can change the physical nature and biodistribution rate. As mentioned earlier, the origin of Exos (host cell type) has an indisputable effect on physicochemical properties. For example, changes in cell membrane phospholipids can subsequently alter final lipid content and type in releasing Exos. These features support Exo hardness, density, diameter, stability, and interaction with non-immune/immune cells [63]. Unfortunately, most previous in vivo studies have been performed on small animal models to assess the efficiency of nanoformulations. Whether and how these nanoformulations can yield similar therapeutic outcomes in other species is the subject of argument. For instance, the eligibility and integrity of synthesized NPs and Exos should be carefully assessed after being exposed to 140,000 dynes/cm^2^ shear stress in the human aorta [64, 65]. It is believed that drug-resistant cancer cells have very prominent biophysical properties such as significant stability which is reflected in their by-products such as Exo. Decoration and manipulation of the Exo surface with different antibody types may change the content, composition, and conformation of PC related to the naïve Exos. In experiments carried out by two research groups, Exos exhibited unpredicted tissue distribution after being incubated with plasma. In this regard, fluorescence-tagged Exos isolated from cancer cells were distributed to different sites with minimum accumulation in the target sites [14, 66, 67]. As a correlate, the underlying mechanisms orchestrating Exo distribution remain unaddressed. In the context of cancer biology, this question is unanswered whether and how cancer cell-related Exos can be influenced via PC formation in plasma and/or other biofluids. It seems that cancer cells can produce Exos with low cargo content like several different miRNAs, siRNAs, inflammatory cytokines, etc. but having similar biological properties. Besides, the distribution of these biomolecules occurs intentionally in a programmable manner. It has been shown that mir-21A containing Exos can polarize migrated macrophages to M2-type phenotype within tumor parenchyma to suppress anti-tumor activity [68–70]. Unfortunately, the paucity of enough pieces of evidence and lack of universal rule has led to ambiguity in the prediction of Exo-PC interaction [14, 71, 72] (Fig. 2). Protein contamination is one of the most challenging issues during Exo collection, especially in terms of allogeneic therapeutic purposes. This phenomenon can increase the risk of allogeneic immune response after the formation of PC around Exos. The collection and isolation of Exos from in vitro settings can yield a similar pitfall. Fetal bovine serum (FBS) is usually used as a protein supplement for cell growth and expansion. Results have shown mild to moderate possibility of xeno-immunization and transmission of zoonotic agents [73]. Besides to aggregation of different growth factor types and other proteins on the Exo surface, genetic materials such as DNA can be adsorbed by Exos inside the culture medium [74]. Albumin, as a major plasma component, can constitute both weak and strong interactions with calf thymus DNA fragments in aqueous phases. Therefore, the residue of DNA fragments on the Exo surface can generate direct DNA-albumin interaction, resulting in an increased Exo hydrodynamic diameter [75]. Like DNA, RNA residues such as miRNAs can be also problematic. Compared to DNA fragments, RNAs are more susceptible to enzymatic activity in serum. Therefore, it is logical to hypothesize that the interaction of RNA with protein plasma can lead to the formation of PC [76]. Along with this claim, FTIR spectroscopy and affinity capillary electrophoresis confirmed the interaction of plasma albumin with tRNA under physiological conditions with no prominent changes in the albumin structure [76]. Other factors like the administration route, loading of the therapeutic cargoes, and surface modification play key roles in the composition of PC around Exos. It has been demonstrated that intraperitoneally administration of several types of NPs can result in the formation of specific PC subsets when compared to other injection ways like intravenous, intrapulmonary, and intra-tracheal routes. As expected, these conditions can affect biodistribution and target delivery [77–79]. It was suggested that a large number of administrated NPs and Exos accumulate in hepatic and pulmonary tissues after intraperitoneal injection while intravenous administration or pulmonary lavage displays the opposite effects. It is believed that hepatic tissue is an important niche for biological barriers and about 30–99% of particles can be quickly eliminated from blood. However, the size, shape, modality (softness and hardness), zeta potential, and surface chemistry can change liver tissue uptake [63].

**Fig. 3:**
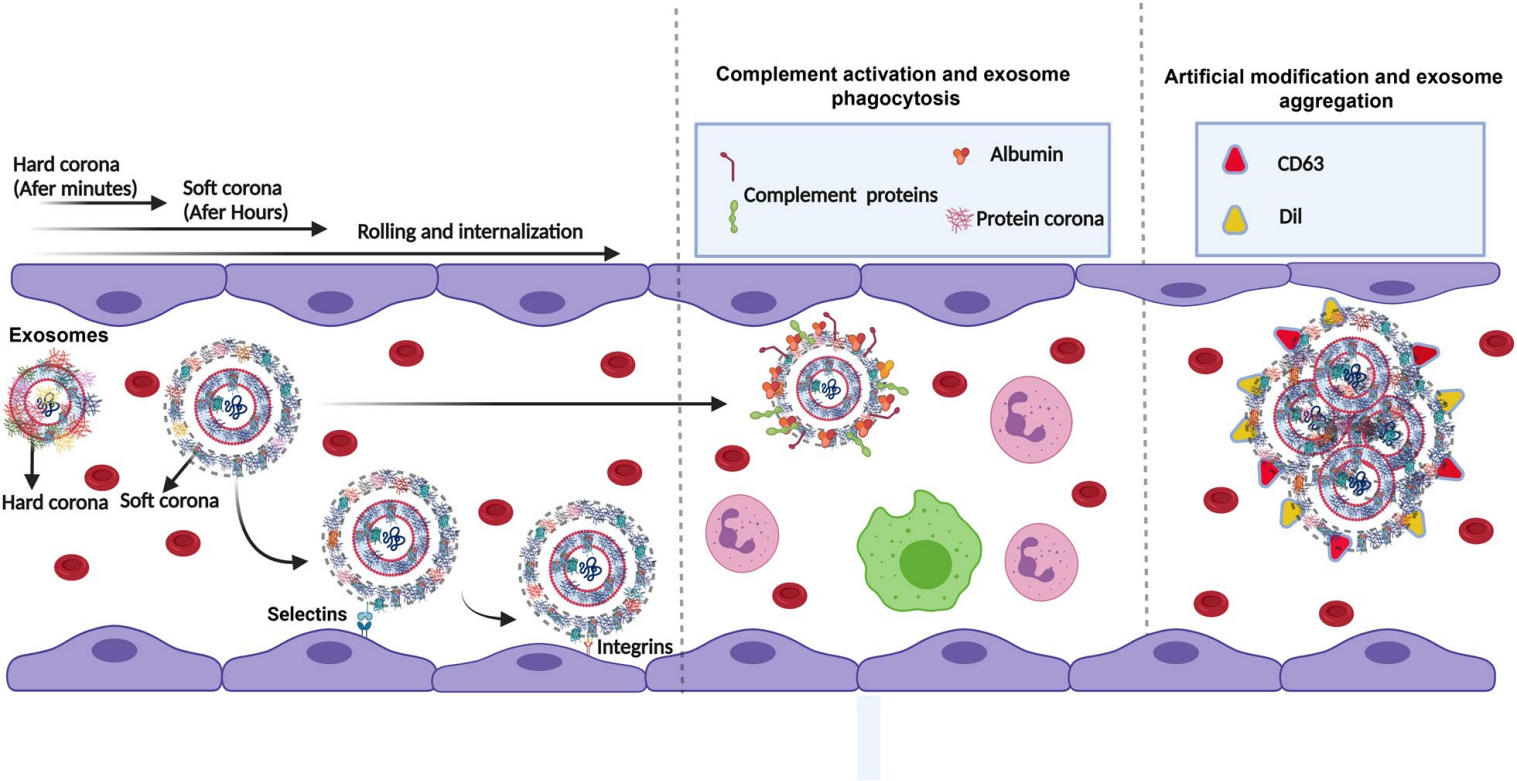
PC formation on the surface of Exos inside in vivo conditions. Both hard and soft PC layers are generated around Exos as a result of non-specific interactions, and ligand-receptor affinity. The binding of different serum proteins on the exosomal surface can affect the hydrodynamic size, biodistribution, colloidal stability, and ligand-receptor interaction between Exos and acceptor cells. The formation of PC around Exos can also lead to the scavenging of these nanoparticles via the reticuloendothelial system. Meanwhile, the circulation time and delivery capacity of Exos are diminished as well. The binding of specific factors such as complement subsets increases the uptake of Exo by immune cells

To date, different arrays of formulations have been used to improve therapeutic efficiency and target delivery of Exos by induction of specific membrane-bound proteins inside the host cells before the release of Exos into the ECM [80]. Such strategies, not completely but in part, can alter the topographical features of Exos when compared to wild Exos [81]. Very recently, it has been indicated that certain plasma proteins including complement factors 3 and 4B, Apolipoprotein A1, B, C3, and E, α component of fibrinogen, immunoglobulin heavy chains of (γ_2_ and γ_4_) are common PC subsets on the surface of Exos, virions, and synthetic NPs upon exposure to the plasma [14] (Fig. 3). The determination of PC around synthetic NPs and virions is relatively applicable compared to Exo PC. In most circumstances, the structure of NPs is not problematic to PC analysis [35]. Similarly, in virions, any changes in the composition of PC can be detected due to known structure and composition. In the case of Exos, the story is so complicated because of extensive Exo heterogeneity in size and component [82]. Of note, any changes in metabolic activity and biological information of originating cells can alter the molecular chemical composition of Exos [83]. Metabolic diversity between cells can lead to the secretion of several Exo types into the blood and unpredictable data related to PC composition [14, 84]. Commensurate with these descriptions, it seems that PC composition is relatively uniform and predictable in Exos isolated from in vitro culture systems. Of course, apparent differences between certain cell types should not be neglected in terms of PC entity [85].

Certain plasma protein subsets such as complement factors 3 and 4B attach to the Exo surface, resulting in robust phagocytosis of opsonized Exos by immune cells. This phenomenon can lead to a reduction of transit time through the blood [14]. Besides, the activation of the complement system triggers secondary inflammatory reactions. It was suggested that the complement system acts as a bridge between innate and adaptive immune system elements [86]. This feature is more highlighted when allogenic Exos are administrated. However, the possible effect of person-specificity in adsorption and activation of the complement system in the allogenic niche needs further investigation. Here, the main question remains unanswered whether specific complement subsets are only physically attached to the Exo surface or enzymatically activated after time. Further molecular investigations are mandatory to examine the attachment of complement inhibitory proteins concurrent with the adsorption of complement factors 3 and 4B. Alterations in the Exo surface complement profile have been indicated under pathological conditions [87]. Previous experiments have shown that following the onset of systemic lupus erythematous, Exos harbor large contents of C3d-opsonized immune complexes while the levels of C3b and C3ib are diminished. It seems that this condition can reduce Exo phagocytosis by immune cells and increase exosomal transit time, leading to prolonged chronic inflammatory conditions [88]. The selective adsorption of complement factors to the Exo surface can be used for immunization purposes since the reduction of C3b and C3ib affects the adjuvant efficiency of Exos in in vivo conditions [89]. Interestingly, the existence of certain complement types such as C3b on the surface of ILVs within the MVBs indicates the possibility of complement system loading on Exo. Thus, a fraction of complement subsets can be loaded onto the Exos prior to entry to blood. It is suggested that C3b-coated Exos participate in immunomodulation via the presentation of antigens to antigen-presenting cells [89]. Despite the existence of several opsonin subsets in PC composition around Exos, Tóth and co-workers did not find monocyte and neutrophil phagocytic activities against Exos decorated with PC. One reason would be related to the existence of immune-elusive mechanisms on the Exo surface that facilitates tolerability and circulation time [90]. For example, the existence of CD47 on the Exo surface can lead to the transmission of “do not eat me” signals toward immune cells [91]. The interaction of this ligand with macrophage membrane glycoprotein SIRPα inhibits the phagocytic activity [92]. Noteworthy, increasing the circulation time by attaching specific proteins or even polymers like PEG cannot be useful. This phenomenon can lead to an increased biodistribution rate without reaching the targeted sites. Even in the case of PEG application, the patient's body produces anti-PEG IgM after administration which subsequently decreases the efficacy of the formulation [93–95]. So, this strategy will not successful, unless the attached anti-opsonin proteins have final landing tissue for specific drug delivery to an organ [96]. Based on recent experiments, it has been proposed that the existence of certain apolipoproteins such as ApoE and ApoB100 on particle surface can increase the transfer rate into the peripheral tissues via using lipoprotein receptors located on the apical surface of endothelial cells [97, 98]. These receptors participate in the transcellular transfer procedure [97, 98]. The attachment of certain PC types could be beneficial in the delivery of decorated particles into the target sites. Data indicated the critical role of lipoproteins in the Exo function [87]. In support of this notion, laboratory analysis of lipoprotein fragments revealed the existence of certain molecular profiles such as S100-A8, LDL-receptor, CD14, and HLA class I, indicating the interfering role of lipoprotein in the dynamic activity of Exos [99]. Whether lipoprotein accumulation on the Exo surface during circulation and exposure to ECM components can alter normal activity is the subject of debate [99]. Previous studies have indicated that Exos are in close contact with ECM components before entry into the blood [100]. This effect can help us to forecast what happens inside the tissue. Therefore, one could hypothesize that the bimolecular formation inside the interstitial matrix is before PC formation in blood in the latter stages.

Based on some facts, artificial manipulation of the Exo surface, not only, can change the composition of PC but also alter the bimolecular distribution pattern. In a recent experiment, it was indicated that partial separation of extra-Exo proteins using size exclusion chromatography blunted in vitro proangiogenic properties [101]. In contrast, isolation via tangential flow-filtration led to the enrichment of the Exo surface with varied factors associated with immunomodulatory and proangiogenic properties. The physical connection with an exosomal surface can increase the stability of angiogenesis factors like VEGF, angiopoietins, etc. from degradation by proteases such as metalloproteinases (MMP-8) [101]. Besides, these features show that Exos can induce specific behavior in the target cells via the adsorption of certain factors in biofluids in addition to the existence of effective convergent luminal contents. Some studies have investigated the tendency of pro-coagulant factors onto the Exo surface [87]. Phosphatidylserine and tissue factor are the main coagulant factors on the surface of platelet Exos that facilitate the assembly of other coagulation cascade members such as VII, IX, X, and prothrombin, resulting in thromboembolic complications [102, 103]. Likewise, coagulation inhibitory factors such as tissue factor pathway inhibitors are also present on the Exo surface [104]. Regarding the critical role of platelets in the coagulation process, it seems that the intensity of coagulation factors in platelets Exos is higher when compared to the Exos from other cell sources [105].

There are some conflicting results regarding the existence of albumin on the Exo surface [14]. Albumin is the most abundant plasma protein and in collaboration with other factors such as fibronectin, complement system, and prothrombin attaches to the Exo surface and generates dense Exos with specific PC [106].  The albumin/globulin ratio (AGR) is significantly decreased after the promotion of pathological conditions [107]. For instance, the AGR level is lower in COPD patients than that of healthy controls because of chronic inflammation and small airways [107]. Notably, it remains unanswered questions whether PC can affect the bioactivity of Exos even under physiological conditions. Are there any mechanisms that Exos can use to decrease the formation of PC when exposed to biofluids? It appears that the development of in vivo models in the least evolved species such as zebrafish helped us to reach a better understanding of Exo biogenesis and PC formation. To the best of our knowledge, there are a small number of studies that explored the biogenesis and dynamics of Exos in less evolved creatures. In zebrafish embryos, the development of pH luorin-labeled CD63 on the Exo surface did not yield statistically significant changes in mean diameter compared to the wild Exos [108]. These features demonstrate that minor molecular manipulations and surface modifications on Exos do not lead to significant changes in physicochemical properties. The production of CP05-modified Exos harboring factors such as M12, RVG, and SP94 increased the uptake rate by acceptor cells [109]. It seems that a load of distinct peptides and molecules on the Exo surface using chemically-linked approaches likely changes surface structure properties. Therefore, caution should be taken in the production of engineered Exos. Protein ubiquitination is an appropriate loading strategy to increase the sorting of target proteins and signaling molecules in the lumen and the surface of Exo using the WW tag and late-domain pathway [110]. In most of the experiments, chemically modified Exos exhibited better delivery efficiency [111]. If we assume that these surface medications can alter the exosomal net charge and other surface features in a favor of PC formation, the uptake efficiency is not diminished compared to wild Exos. Of course, the possibility of PC formation should not be overlooked in terms of autologous and allogenic Exos. It has been indicated that Exos with mismatched MHC-I and II can promote T cell-related responses [112]. Although Exos display very low levels of MHC and these molecules possess different ranges of peptide-binding capacities, possibly changing the dynamic interaction of exosomal surface molecules with serum factors [113, 114]. Besides, some classes of MHC subsets (mainly MHC-III) can recall complement system effectors. Due to the lack of reports regarding the existence of the MHC-III system on the Exo surface, it seems that MHC-II and especially MHC-I are more important players in the attachment of Exos to protein fragments in serum [113]. Concerning immune system cell types, recent works have indicated that Exos released by dendritic cells, so-called dexosomes, have higher MHC-I and II contents compared to Exos isolated from non-immune cells [115]. Irrespective of the activation of phagocytic mechanisms and cellular immunity in Exos with mismatched MHCs, the extent, and intensity of PC formation around allogenic and xenogeneic Exos should be investigated in future studies. Several experiments have revealed similar behavior related to endogenous and exogenous Exos in the terms of tethering, and rolling on the luminal surface of vasculature endothelial cells [116]. These features may point to the fact that if the PC formation is likely around both Exo types, variety in corona protein subsets does not alter Exos behavior [116]. Of note, the behavior and dynamic activity of GFP-tagged Exos are a little different inside the blood when compared to in vitro systems. Monitoring GFP-tagged Exos in zebrafish revealed irregular Brownian motion outside the vasculature system compared to the circulation system [108]. It is postulated that attachment or aggregation of PC components on the exosomal surface in biofluids with lower velocity or circulation could be problematic. In line with these descriptions, exogenous Exos exhibited different trafficking patterns following using the same injection method [117]. As a separate note, the formation of PC and the increase of hydrodynamic radius promotes the aggregation of nano-sized particles in in vivo conditions [37]. Not surprisingly, the hydrodynamic diameter of Exos is inversely associated with biodistribution properties. Based on a recent experiment, trypsinization can reduce coronal layer and hydrodynamic diameter compared to the wild Exos. These changes were more evident in protein kinase-treated Exos [118] (Fig. 2). Based on enzyme activity, trypsin acts on lysine and arginine motifs and does not digest anchored segments of bound PC while protein kinase can digest hydrophobic, aliphatic, and aromatic amino acids, resulting in the formation of thinner coronal layer [118]. Based on these results, the variety in the PC subsets can result in Exo heterogeneity in mean diameter. It is worth mentioning that the existence of different enzymes inside serum such as metalloproteinases can affect the hydrodynamic diameter of Exos in favor of motility and biodistribution [119]. Of course, PC formation surrounding NPs diminishes surface energy via non-specific interactions and thus decreases affinity to the cell membrane surface [120]. Importantly, any changes in physiological pH and temperature, as seen in pathological conditions, also alter the 3D folding capacity of the soft corona layer. These differences are consistent with the fact that equilibrium between unfolded and folded states can affect further interaction of the attached protein with serum proteins [121].

## Impact of various pathological conditions on PC component

In accordance with previous investigations, it has been accepted that the profile of PC can be substantially changed according to slight variations in blood plasma [122]. The concept of personalized PC has been introduced when the composition of absorbed serum proteins onto nanomaterials exhibited variations after incubating with serum proteins of different individuals who experienced different pathological conditions. In another word, analyzing the plasma proteins under several pathological conditions confirmed the alteration in type and 3D conformation of PC subsets [55]. For instance, liposomes exposed to pancreatic cancer sera adsorbed proteins with less negative charge compared to breast cancer sera. Further investigations indicated that the profile of PC in pancreatic cancer is mostly composed of immunoglobulin alpha (IgA) and immunoglobulin gamma (IgG) [123]. Some pathological conditions result from conformational changes of various types of proteins which are known as proteinopathies diseases such as amyloidosis. The conformational changes of specific proteins under pathological conditions lead to the aggregation of misfolded proteins. Subsequently, these changes might affect the interaction of serum proteins with NPs in blood flow. It was confirmed the existence of distinct proteins during certain proteinopathies could change the profile of PC [124]. The incubation of graphene oxide sheets with plasma from patients with different pathological conditions including healthy, cancer, hypofibrinogenemia pregnancy, diabetes, fauvism, and rheumatism revealed significant variations in hard PC compositions. In another word, several components of PC might appear or disappear in plasma samples of volunteers with different pathological conditions [40].

## Potential strategies to improve therapeutic efficiency of Exos through targeting PC

Despite the possibility of PC formation on the exosomal surface, different experiments have shown that Exos are suitable drug delivery carriers. However, the main question remains unanswered whether the formation of PC can be problematic or it does not affect the dynamic biodistribution and target delivery. If the PC layer surrounds the external surface of Exos after being exposed to biological fluids, how Exos can circumvent these interfering effects? The possible answer to this question may be addressed by an experiment conducted by Warren et al. in 2020. Indeed, they introduced the term “threshold for administered NP number [125, 126]. To be specific, hepatic macrophages namely, Kupffer cells which are about 80–90% of the total macrophage population display threshold saturation [127]. In response to eliminating circulating NPs, these cells can uptake these particles until reaching saturation. Despite all the problems in the field of PC formation and its role in targeting, combination therapies seem to be appropriate strategies. Up to now, there are two strategies have been introduced, including pre-incubation of Exos with plasma (in most cases healthy derived plasma), and then surface modification can yield very good results [128–131]. One reason would be that the sharp reduction of the surface physical forces to a surface in NPs is no longer able to adsorb other proteins. Besides, the application of some therapeutic agents to suppress the complement system, especially C3, Cq1, etc. plays an important role in the labeling of NPs and Exos and their clearance rate [132]. However, the authors suggested that the best and simple strategy for using Exos and other nano-carriers is to find their biodistribution rate with any properties before loading specific biomolecules. Besides, NPs and Exos should be engineered in an autologous manner for personalized medicine purposes.Therefore, steady-state PC formation and soft-to-hard layer ratio are mighty altered during the onset of several pathologies. Perhaps, the behavior of Exos under pathological conditions can be different in comparison with what happens under physiological conditions. In a better word, the affinity of exosomal ligands with cell receptors and other serum proteins can be changed in varying degrees. So, it is mandatory to apply sophisticated design methods for the preparation of natural and synthetic NPs.

## Conclusion

In conclusion, there is ambiguity related to the underlying mechanism of PC formation around Exos inside the body. Considering the ability of wide ranges of molecules to adsorb onto the Exo surface and the existence of person-to-person variations in serum proteins, the coronal thickness is thought to be significantly different, affecting exosomal kinetics, biodistribution, docking, and internalization. If assumed that the major constituents of PC are common serum proteins thus it is logical to hypothesize that decoration of allogenic immunogens with PC can protect administrated Exos from the immune system. Of course, the activity of other pro-inflammatory cytokines should not be neglected. It seems that the heterogeneity of PC and other factors attached to the Exo surface is high due to the complexity of the exosomal wall with several ligands when compared to synthetic NPs. In general, it can be said in simple and easy words that the formation of PC around Exos can change the physicochemical properties and possibly target capacity. Because of intraspecific genetic diversity from person to person, it seems that the entity and loading capacity of PC can be very diverse due to heterogeneity in donor Exos and serum protein profile in recipients. Irrespective of these diversities, the occurrence of pathological conditions can intensify these complexities. It is suggested that future studies focus on the detection of PC composition during physiological and pathological conditions.

## Data Availability

Not applicable.

## Abbreviations

AGR: Albumin/Globulin ratio
ApoE: Apolipoprotein E
COPD: Chronic obstructive pulmonary disease
C3b: Complement component 3b
ELISA: Enzyme-linked immunosorbent assay
Exo: Exosome
Exos: Exosomes
ECM: Extracellular matrix
EVs: Extracellular vesicles
FBS: Fetal bovine serum
AuNPs: Gold nanoparticles
GFP: Green fluorescent protein
HSV1: Human Herpes Simplex Virus-1
IgG: Immunoglobulin G
ILVs: Intraluminal vesicles
LC–MS/MS: Label-free shotgun tandem mass spectrometry
MVBs: Multivesicular bodies
NPs: Nanoparticles
NO: Nitric oxide
PEG: Polyethylene glycol
ROS: Reactive oxygen species
TME: Tumor extracellular microenvironment
